# Effect of using spirulina algae methyl ester on the performance of a diesel engine with changing compression ratio: an experimental investigation

**DOI:** 10.1038/s41598-022-23233-6

**Published:** 2022-10-28

**Authors:** Mohamed F. Al-Dawody, Duraid F. Maki, Khaled Al-Farhany, Mujtaba A. Flayyih, Wasim Jamshed, El Sayed M. Tag El Din, Zehba Raizah

**Affiliations:** 1 Department of Mechanical Engineering, University of Al-Qadisiyah, Al-Qadisiyah, 58001 Iraq; 2 Department of Mechanical Engineering, Babylon University, Babylon, Iraq; 3 Biomedical Engineering Department, Al-Mustaqbal University College, Hillah, Babylon Iraq; 4 Department of Mathematics, Capital University of Science and Technology (CUST), Islamabad, 44000 Pakistan; 5 Electrical Engineering, Faculty of Engineering and Technology, Future University in Egypt, New Cairo, 11835 Egypt; 6 Department of Mathematics, College of Science, Abha, King Khalid University, 62529 Abha, Saudi Arabia

**Keywords:** Mathematics and computing, Physics

## Abstract

Diesel engine characteristics were investigated experimentally while adding different concentrations of third generation biodiesel spirulina algae methyl ester (SAME). Three volumetric blends of SAME are added to standard Iraqi diesel, namely 10% SAME, 20% SAME, and 30% SAME. The properties of the fuels were found according to the American Society for Testing and Materials standards (ASTM). Experimental work was conducted on a single-cylinder diesel engine under variable load and compression ratio. Three compression ratios are used, starting from 14.5, 15.5, and 16.5. Based on the results obtained, the presence of SAME along with diesel caused an increase in Brake specific fuel consumption (BSFC), carbon dioxide (CO_2_)_,_ and nitrogen oxides (NO_x_) while decreasing both brake thermal efficiency (BTE) and exhaust gas temperature (EGT). Hydrocarbon (HC) emissions decreased by 7.14%, 8.57%, and 10.71%, for 10% SAME, 20% SAME, and 30% SAME, respectively, compared to the original neat diesel fuel. The dramatic carbon monoxide (CO) emission reduction was at full load point. The addition of SAME from (10 to 30)% reported a decrease in CO by (6.67–20)%. NOx, as well as CO_2_ emission, are increased as a result of SAME addition. The compression ratio change from (14.5/1 to 16.5/1) led to increased BTE, NOx, and decreased BSFC and all carbon emissions. The experimental results are validated with other studies' findings, and minor divergence is reported.

## Introduction

As the global population rises and the average standard of living rises, so does the need for energy. In order to keep up with this surge in energy consumption, fossil fuels have been increasingly used, which has led to the significant issue of fossil fuel depletion and a rise in the price of fossil fuels. Fossil fuel reserves are quickly running out, which makes it necessary to explore for alternate fuels like biofuels. It is known that fossil fuels, like crude oil, take years to produce, and with the present pace of exploitation, they are expected to run out soon^1^. Furthermore, the toxic compounds cause smog to form undesirable climatic changes. Furthermore, their use exacerbates significant environmental impacts like the growth of greenhouse gases, the depletion of the ozone layer, the destruction of forests, the production of acid rain, the formation of photochemical smog, and the eutrophication of water supplies^2^. Therefore, there has to be a rapid exploration of renewable energy options that are affordable, sustainable, and easily accessible^3^.

Biodiesel and alcohols have been the most studied alternatives to diesel fuel, and their potential to reduce diesel fuel use and pollution emissions have attracted the attention of many scientists^4^. The use of biodiesel made from vegetable oils and animal fats as a viable substitute for diesel fuel made from petroleum is an active area of study. Biodiesel made from both edible and non-edible vegetable oils shows promise in this area. This is because they can produce food near home, sometimes even in arid territory. In general, most writers have reported positive outcomes from cooking with vegetable oils such as coconut, soybean, castor, and sunflower^5–8^.

The world's energy needs can be met most effectively via the utilization of renewable power sources due to their great potential and large quantity^9^. In addition, it has recently gained importance due to its potential to replace petroleum derivatives, which are predicted to be depleted within a century. Since the former is better for the environment, biodiesel fuel is increasingly used as an alternative to fossil fuels to reduce harmful emissions. Biodiesel is an oil-based fuel composed of several mono-alkyl esters^3^. Due to the carbon being permanently sequestered in the exhaust, this atmospheric fuel has zero net carbon emissions. Furthermore, since it does not contribute to increased atmospheric carbon dioxide (CO_2_), biodiesel usage mitigates the greenhouse impact^10^. The oxygen in biodiesel reduces its heating value, but it has many other uses. Despite this problem, using biodiesel fuel in CI engines has decreased HC, CO, and smoke emissions while also significantly increasing NOx capacity^11,12^. Animal and vegetable oils are both commonplace in the biodiesel industry as feedstock. Castor oil can be used as a biodiesel feedstock because it doesn’t need much energy and has no edible oils^13^. Castor plants may thrive without fertilizer in the hot, humid climate of the tropics^14^.

Researchers from around the globe have tried out biodiesel as a diesel engine fuel alternative in various tests.

Rajak et al.^15^ used the Diesel-RK engine simulator to conduct a numerical study of diesel engine characteristics. The diesel fuel compression ratio for the engine ranges from 16.5/1 to 18.5/1. Maximum load test trials were done with an optimum compression ratio of 17.5 and compared to numerical findings. The products reduced thermal efficiency by 2.73%, torque by 6.66%, exhaust gas temperature by 1.6%, carbon dioxide by 6.1%, and nitrogen oxide by 0.5% at full load and constant speed. However, SFC rose by 6.4% compared to regular diesel when CR was raised to 17.5. Naresh et al.^16^. Researched the impact of changing the EGR control setting from 5 to 20% on the characteristics of a diesel engine.

Under different loads, the thermal brake efficiency of engines driving on biodiesel or a blend of biodiesel and diesel was clearly lower than that of diesel-powered engines. As the proportion of blended fuels grows, energy needs drop because of the lower calorific values of the combined fuels. As EGR is used, NOx and HC emissions are reduced. Increases in the exhaust gas recirculation rate reduce emissions and boost performance. EGR at 15% is recommended to maximize fuel performance and emission characteristics. Rajak et al.^17^ tested a normally aspirated diesel engine under different loads to see how the ratio of Spirulina microalgae biodiesel to conventional fuel changed performance. Results indicated a decrease in cylinder pressure and thermal efficiency compared to diesel fuel. NOx, PM, and smoke are reduced by 4.9%, 20.7%, and 5.4%, respectively. Satputaly et al.^18^ transesterified the oil extracted from chlorella microalgae to create biodiesel. A 5.2 kW diesel engine was used to evaluate the biodiesel produced for combustion, performance, and emission. There was a 3.09% drop in thermal brake efficiency. The results indicated that compared to diesel, methyl ester microalgae oil resulted in lower emissions of CO, HC, NOx, and smoke opacity. Blends of microalgae oil extracted using soxhlet, which has a viscosity eight times that of diesel, and sunflower oil were created in the following proportions: Mixing 5 ml of algae oil with 95 ml of sunflower oil (5:95), 10 ml of algae oil with 90 ml of sunflower oil (10:90), and 15 ml of algae oil with 85 ml of sunflower oil (15:85) will decrease the viscosity by 5, 10, and 15 ml, respectively. Lastly, methanol was used in a two-step transesterification process to turn the above mixture into biodiesel. Additionally, three types of biodiesel were mixed with regular diesel at a ratio of 10:90 ml (B10A%, B10B%, and B10C%). The research included an analysis of both chemical and physical characteristics. The produced biodiesel conforms to ASTM standards in terms of its quality. The results showed that B10C% biodiesel has a 2.77% higher brake thermal efficiency than regular diesel. When comparing B10C biodiesel to diesel, volumetric efficiency is improved by 1.5% Sankar et al.^19^.

El-Baz et al.^20^ reported biodiesel formulation from microalgae oil; specifically, blends of 10 and 20% biodiesel were made. The investigated mixes had many of the same physical and chemical characteristics as diesel fuel. Specific fuel usage, exhaust gas temperature, and thermal efficiency improved using B20 biodiesel instead of diesel or B10. When compared to B10 and diesel, B20 emits much less pollution. Rajak and Verma^21^ studied the effects of microalgae biodiesel (B20) in an emulsion fuel on the characteristic of a diesel engine. The motor may be set to one of three different speeds. The diesel engine can operate on B0, B20, or B100 in full load conditions. The findings revealed a decrease of 0.5% in indicated thermal efficiency, 6.2% in nitrogen oxides, 1.63% in cylinder pressure, 2.6% in smoke emission, and 1.2% in brake thermal efficiency. At 1500 rpm and full load, the peak heat release rate, specific fuel consumption, carbon dioxide emissions, and ignition delay duration all increased by 5% with B20. The outcomes were compared to experimental findings.

Murthy et al.^22^ cultivated Chlorella Vulgaris algae for biodiesel generation in watery environments. To examine the pollution, combustion, and performance characteristics of diesel engines, the scientists ran a series of experiments using varying mixes of algal biodiesel and diesel fuel and various loads. Since B20 has a different heating value than regular diesel, it uses more fuel per mile driven on the brakes. The emission levels of NOx, CO, and hydrocarbons have decreased.

Mahamudul et al.^23^ as mentioned biodiesel and its mixes increase fuel consumption and reduce small amounts of hazardous emissions such as hydrocarbons, particulate matter, and carbon dioxide. This results in higher NOx emissions. As the plants use CO_2_, it is possible to disregard emissions. It was shown that diesel engines could use biodiesel as an alternative fuel, which is good for the environment and the world's limited resources. Makarevičienė et al.^24^ tested biodiesel using 30% algal oil methyl ester in a VALMET 320 DMG diesel engine aboard the ship. A study indicated that compared to using diesel fuel, using B30 lowered smoke exhaust emissions by 10–75% and hydrocarbon (HC) emissions by 5–25%. When the engine was run on B30 instead of diesel, the thermal efficiency went up by 2.5–3%. Mathimani et al.^25^ examined the suitability of biodiesel generated from microalgae Chlorella Vulgaris. Testing was conducted on biodiesel-diesel blends to determine their performance and emission characteristics in a single-cylinder diesel engine running at different loads (0%, 50%, 75%, 100%). Compared to standard diesel, B50-powered engines produce 102 ppm lower hydrocarbon emissions and 1% less carbon monoxide. The findings indicated a 6.1% decrease in CO2 and a 376 ppm decrease in NOx.

Reddy et al.^26^ Biodiesel made from Schizochytrium microalgae oil was discussed as a potential replacement fuel for conventional diesel engines. The combustion, performance, and emission characteristics of diesel engines fuelled by microalgae biodiesel and their mixtures were studied. Biodiesel characteristics were found to be in accordance with ASTM specifications. Biodiesel engines were shown to have comparable performance and efficiency to their fossil fuel counterparts, so their use was approved. Haik et al.^27^ raw algal oil and its methyl esters were tested in a diesel engine as part of an experiment. Heat dissipation, along with parameters, were all investigated. Because of its similarities to diesel fuel, algae oil methyl ester has been successfully used to power diesel vehicles. However, it somewhat reduced the engine's output torque and increased the combustion noise. Some parts of the engine design, like the compression ratio and when the fuel is injected, can be changed to boost the engine's power and quiet down the noise of combustion. Patel et al.^28^ Biodiesel produced from Indian microalgae was evaluated for its viability. A diesel engine was used to assess the performance of algae biodiesel blends with fossil fuel concentrations of 10%, 15%, and 20%. Brake-specific fuel consumption and carbon monoxide emissions are said to have dropped considerably. In addition, NOx emissions rose dramatically.

Diesel fuel could probably be mixed with algae-based biodiesel to reduce the amount of fossil fuels used while keeping the engine’s performance automobile industry is primarily concerned with engine performance, emissions, and roughness. A Ricardo E6 single-cylinder indirect injection engine was used to conduct comparative experiments between three different biofuels and regular diesel. The algal methyl ester AME is one of the three fuels utilized. In the first series of tests, the engine torque (load) varied between 0.5 and 15 Nm, while the other variables included injection time, engine speed, and compression ratio. The injection times in the second trial varied from (20°) to (45°). BTDC. Due to the wide range of biofuel properties, the results showed that the fuel engine load and injection timing might need to be changed. (Ospina et al.^29^).

The extraction of biomass from diverse feedstocks, such as vegetable oils, animal fats, and other waste sources, has been the subject of much research over the last few decades. In this work, an approach has been made to determine how adding 10, 20, and 30% SAME to 90%, 80%, and 70% Iraqi diesel, respectively, affects the performance of a diesel engine under varying loads and compression ratios. The main novelty in this work is the transesterification of algae bio-oil into biodiesel and successfully tested in a diesel engine.

## Biodiesel production and specifications

**Figure 1:**
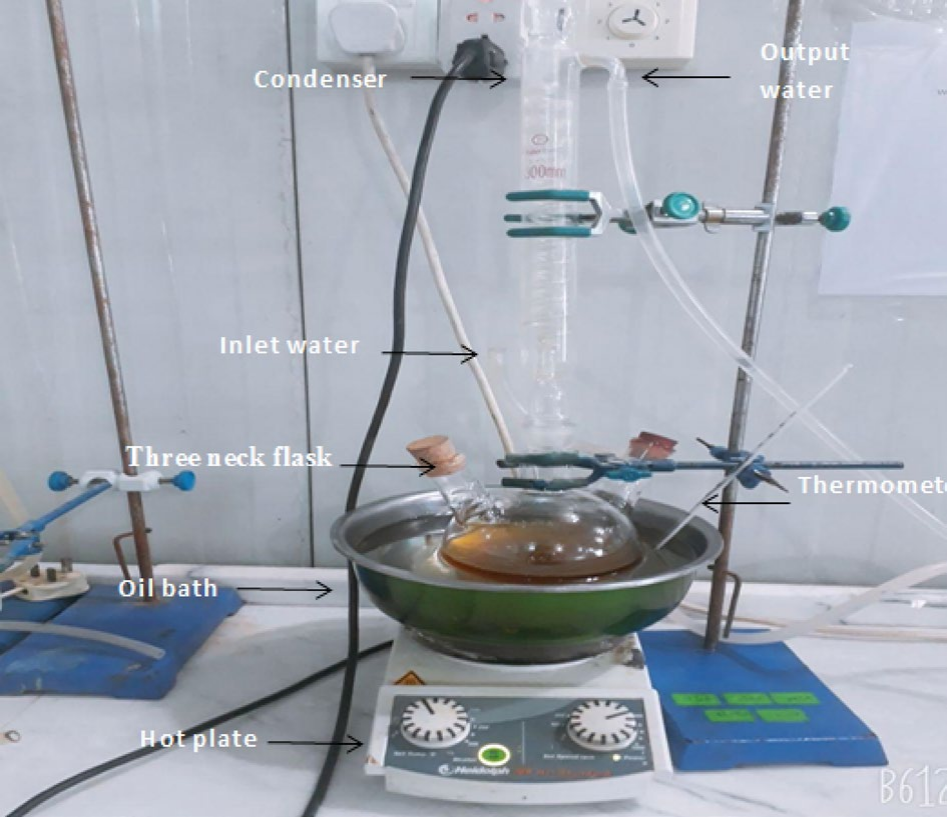
Biodiesel production kit^30^.

In this research, a bio source called Spirulina species was attempted to be converted into biodiesel. It is transformed utilizing the transesterification procedure into Microalgae oil methyl ester. A test was performed on each physical and chemical property, and the results were compared to ASTM standards. Additional experimental tests were conducted to assess the performance and emission characteristics of the fuel that was produced. The previous paper discusses oil microalgae collecting, oil extraction, and SAME manufacturing^30^. Making biodiesel from spirulina algae oil requires the transesterification process described in Eq. (1)^31^. The biodiesel formation process is carried out in a 1-l three-necked flask (see Fig. 1).1

Table 1 presents the properties of the proposed biodiesel (SAME) blends & pure Iraqi diesel. The current experimental work is conducted on a single-cylinder diesel engine; its technical data.

**Table 1:** Iraqi diesel and SAME biodiesel characteristics.

Property	Iraqi diesel	10% SAME	20% SAME	30% SAME	ASTM D6785 standard
Chemical formula	${\mathrm{C}}_{13.77}{\mathrm{H}}_{23.44}$	${\mathrm{C}}_{14.01}{\mathrm{H}}_{24.81}$ O_0.19_	${\mathrm{C}}_{14.22}{\mathrm{H}}_{24.55}{\mathrm{O}}_{0.4}$	${\mathrm{C}}_{14.44}{\mathrm{H}}_{25.11}{\mathrm{O}}_{0.6}$	C_12_–C_22_
Density (kg/m^3^)	830	834	837.8	842.7	860–900
Viscosity (Pa s)	0.002241	0.00245	0.002658	0.002867	0.0019–0.0006
Heating value (MJ/kg)	45.836	45.144	44.4528	43.7612	36–39
Cetane number	53.4	53.045	52.69	52.335	> 47
Molecular w. (g/mol)	190	195.984	201.9688	207.9532	–
Surface tension (N/m)	0.028	0.028124	0.028248	0.02837	–
Fire point (C)	116	111	105	100	–
Flash point (C)	56	55	54	53	–
Cloud point (C)	− 5	− 3.9	− 2.8	− 1.7	–

The Kirloskar TAF-1 details data are given in Table 2.

**Table 2:** Kirloskar TAF-1 information data^32^.

Engine	4-Stroke, diesel engine, single cylinder
Bore	80 mm
Stroke	110 mm
The cylinder capacity	0.55264 l
The compression ratio	Variable (14.5–16.5)
Rated power	3.7 kW, 1500 rpm
Orifice diameter	0.15 mm
Injection pressure	160 bar

## Experimental work

**Figure 2:**
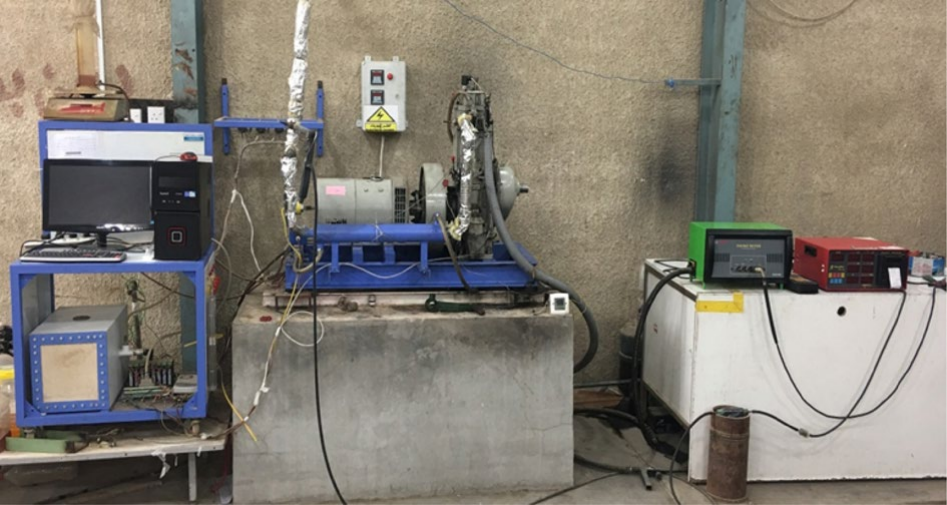
Engine test rig structure.

**Figure 3:**
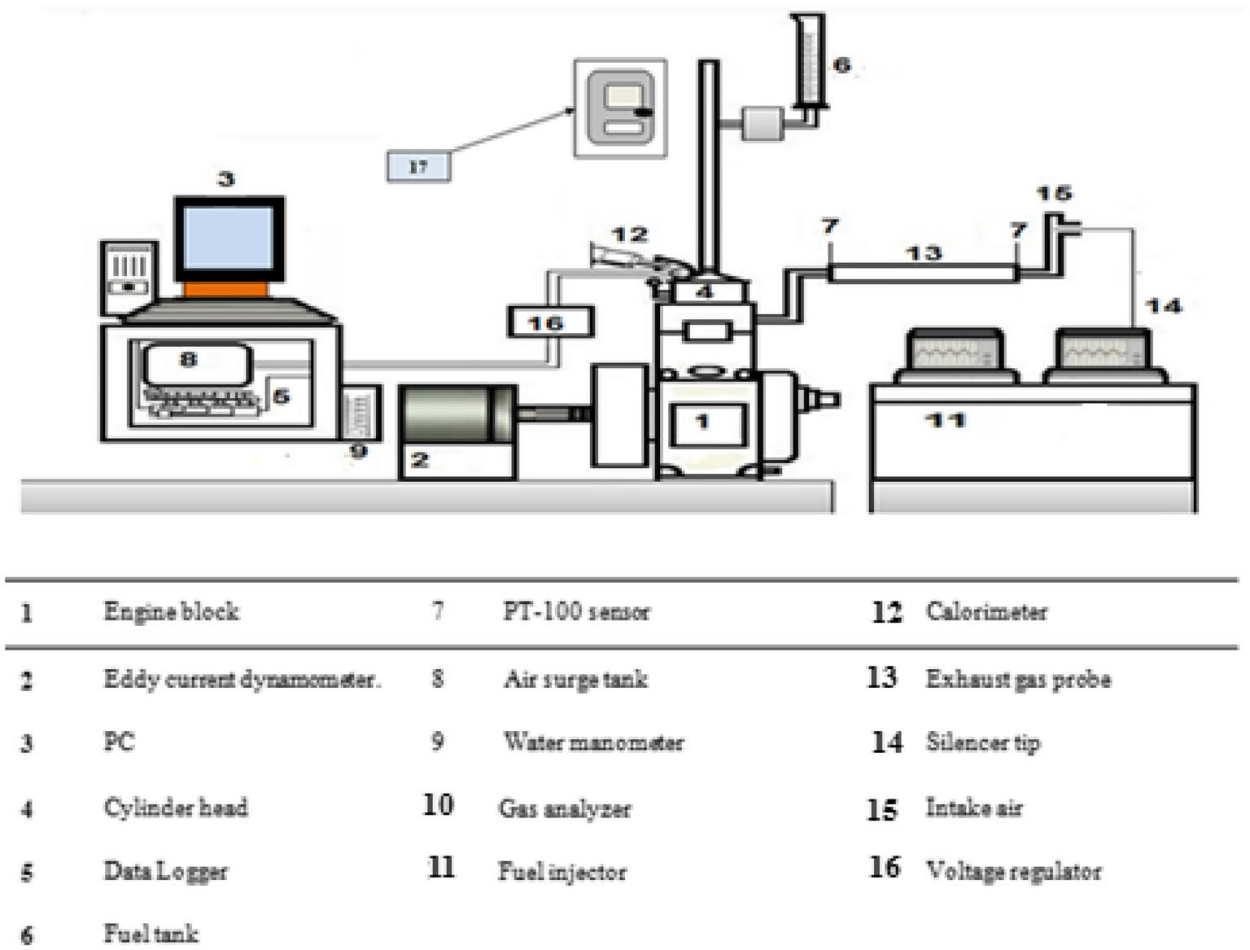
Schematic arrangement of engine step.

The experimental test rig includes a diesel engine, an eddy current dynamometer as the loading system, a cooling water system, and some sensors and instruments integrated with a computerized data acquisition system. Figure 2 is a picture of the engine and its parts, and Fig. 3 is a diagram that shows how the engine works and how it works.

Gas analyzers’ accurate operating ranges are shown in Table 3. The engine operates at a constant speed of 1500 rpm, burning diesel and one of three study samples consisting of 10% SAME, 20% SAME, or 30% SAME. The load is varied from 0 to 3.7 kW in 0.925 kW increments. The data is compared to that obtained from pure diesel. After each test, the prepared sample of fuel is disposed, and the tank (one liter plastic flask) is washed and dried to make it ready for the next test.

**Table 3:** Parameter’s precision and uncertainty.

Measurement	Accuracy	Uncertainty%
Speed	± 15 rpm	1
Time	± 0.8 s	0.2
Temperature	± 1.5 °C	0.1
CO_2_	± 0.1%	0.2
CO	± 0.1%	0.2
HC	± 1 ppm	0.2
NOx	± 1 ppm	0.2

### Uncertainty analysis

Accidental mistakes occur when readings are measured for an experiment. A logical mistake frequently occurs as a result of inaccurate measurement instrument calibration. Any experimental testing must include an assessment of uncertainty. Uncertainty evaluation is carried out by repeating any experiments 3 to 4 times and taking the standard deviation to the measured values. Uncertainty values are evaluated. The uncertainty of any experimental measurement is calculated based on Kline and Mc.Clintock method^33,34^. Table 3 shows the percentage of the uncertainty of the measured parameters. The formulae used in evaluating the uncertainty are the following.

2 $$\Delta R = \left( {\frac{\partial R}{{\partial X_{1} }}\Delta X_{1} + \frac{\partial R}{{\partial X_{2} }}\Delta X_{2} + \frac{\partial R}{{\partial X_{3} }}\Delta X_{3} + \cdots + \frac{\partial R}{{\partial X_{n} }}\Delta X_{n} } \right)^{0.5} ,$$

where ΔR is the total uncertainty of the experimental result. R-main function and relates to variables x_1_,x_2_…xn., Δx_1_, Δx_2_…Δx_n_—Independent variable uncertainties. Based on the study, overall uncertainty is determined within the limit of 4%

## Performance calculation

When ten cubic centimeters (cc) of fuel is used, the fuel consumption time indicator will go into an automated mode and record that amount of time. It is possible to calculate the TFC from this data^32^;

3 $$TFC = \frac{q}{{t_{f} }}\rho_{f} ,$$

where q—The amount of fuel utilized is equal to 0.00001 m^3^, t_f_—Time is taken for 10 (cc) of fuel consumption (s). From Eq. (3), the brake-specific fuel consumption (BSFC) can be calculated:

4 $$BSFC = \frac{TFC}{{BP}}.$$

Multiplying the BSFC by the fuel's lower heating value yields brake-specific energy consumption (BSEC).

5 $$BSEC = BSFC \times Q_{LHVB} ,$$

where the heating value for the blended fuel is given by:

6 $$Q_{LHVB} = \left( {(1 - SAME\% )Q_{LHVDF} + ASME\% \times Q_{LHVSAME} } \right).$$

Lastly, the thermal brake efficiency is computed using the following formula:

7 $$BTE = \frac{3600}{{BSFC \times Q_{LHVB} }}.$$

## Results and discussion

### Validation

Data from experiments is checked against the findings of other studies to ensure their accuracy. Diesel is used as a baseline because it is consistent across different types of literature and because the rules that apply to diesel also apply to other fuel mixtures. Data from two different studies by the groups Rajak et al.^35^, Prakash et al.^36^, and Dassari et al.^37^ are used for validation. The impact of various biofuels on diesel engine characteristics was studied quantitatively by Rajak et al.^35^. Prakash et al.^36^ and Dassari et al.^37^ experimented to find out how CME and bio-ethanol change the way diesel engines work. Full load, 1500 RPM engine speed, and a compression ratio of 15.5 are all typical operating conditions.

**Figure 4:**
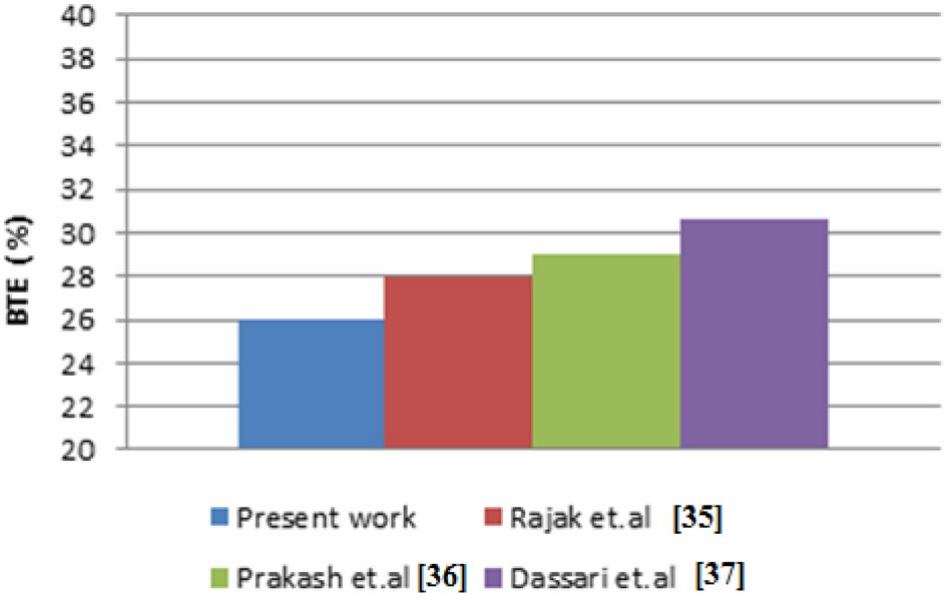
Validation of BTE with various studies.

Figure 4 illustrates the validation of BTE by comparing the current study’s findings to those of the cited literature. The range of BTE values is between 26 and 30%. The findings from the comparison are consistent with one another.

Furthermore, the present work results are also validated with the findings of Subramaniam^38^ which deals with Algae biodiesel and diesel. The HC, CO emissions as well as EGT are reduced as a results of adding SAME, while other emissions like CO_2_ and NOx are increased. The comparison is encouraging with a slight difference.

### Examination of the performance and emissions parameters

**Figure 5:**
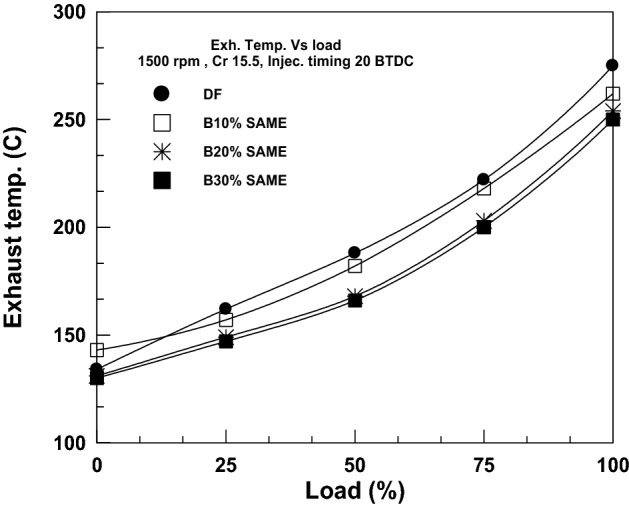
Change in EGT with brake power.

The evolution of the exhaust gas temperature under varying loads for diesel and SAME mixes is shown in Fig. 5. A corresponding rise in exhaust temperature reflects increases in total energy input. Diesel has the highest temperature readings, followed by SAME mixes. Because of the high viscosity and volatility of SAME, the exhaust temperature drops as its proportion rises. 10% SAME reduces EGT by 4.72% compared to plain diesel, 20% SAME reduces EGT by 7.63%, and 30% SAME reduces EGT by 9.09%.

**Figure 6:**
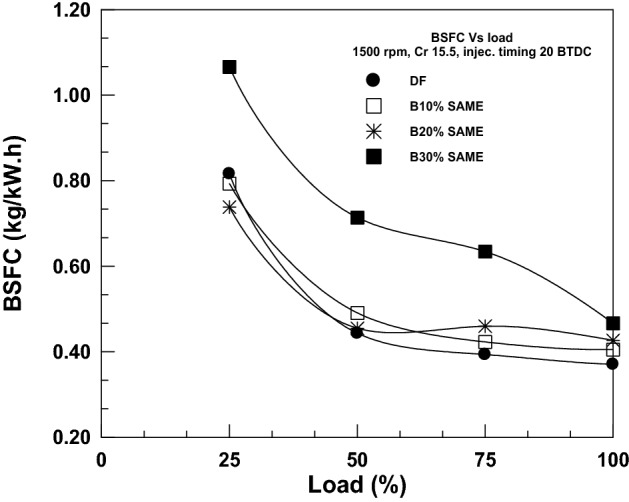
Change in BSFC with brake power.

Figure 6 displays the BSFC load-dependent fluctuation over a range of SAME mix ratios. As the load on the engine grew, the BSFC was found to decrease. When an engine is overworked, the rate of rising in braking power is substantially larger than the rate of increase in fuel consumption, which explains the fall in output. On top of that, since biodiesel SAME is an oxygenated fuel, it promotes even more thorough combustion and has a lower impact on BSFC. The value of BSFC was determined to be 0.738 kg/kWh with 20% SAME at 25% load and 0.426 kg/kWh at full load. As CME blending was increased, it was observed that BSFC rose because biodiesel has a lower heating value, higher density, and higher viscosity than DF. At full load, BSFC increased by 9.27%, 15.14%, and 24.84% for 10%, 20%, and 30% SAME, respectively, above DF. Different writers cite the same findings in their earlier work yet^37,39^.

**Figure 7:**
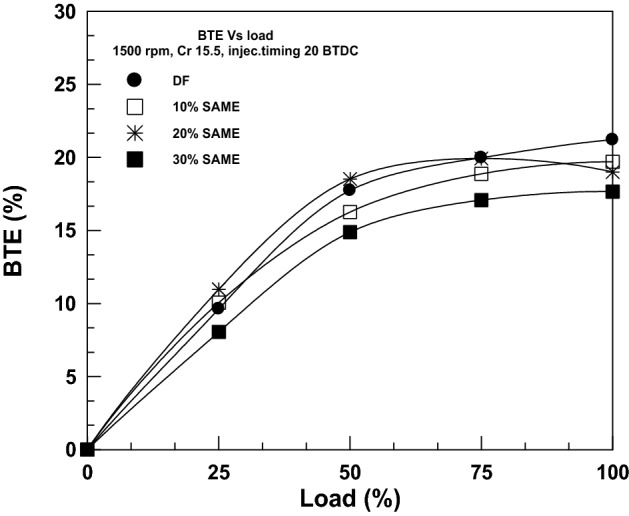
Change in BTE with brake power.

The change of BTE with load at various SAME mixing ratios is reported in Fig. 7. It was shown that the BTE increased with increasing engine load for all tested fuels. The direct implications of BSFC on brake thermal efficiency (BTE) for the engine cycle cause BTE to decline as SAME blending ratios increase. According to Eqs. (2) and (5), the BTE directly results from the BSFC and the lower heating value. Thus, more biodiesel means a higher BSFC and fewer biodiesel transesterification equivalents (BTE). The findings revealed that the BTE dropped by 7.08% for 10% SAME, 10.44% for 20% SAME, and 16.769% for 30% SAME compared to DF under full load. The same findings were obtained in prior studies^40–42^.

**Figure 8:**
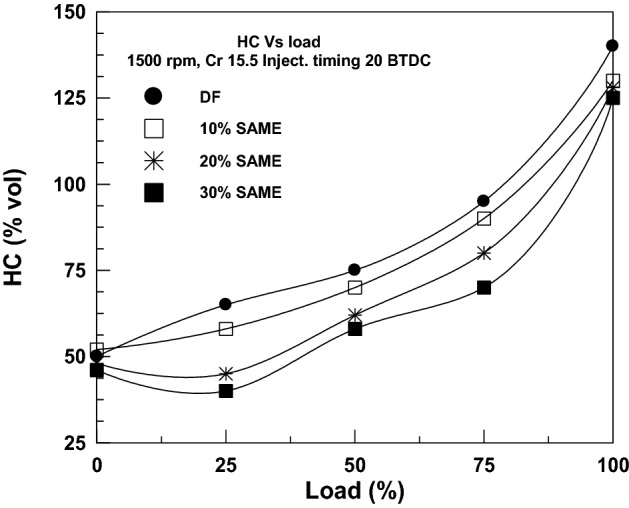
Change in HC with brake power.

The relationship between HC emissions and load (in terms of braking power) is seen in Fig. 8. When the fuel-to-air ratio is raised, the load must also be introduced. Because of their oxidizing properties, HC emissions are reduced when biodiesel blends are increased. Doing so may improve combustion by increasing the number of oxygen molecules in the combustion chamber. Compared to the original plain diesel fuel, HC emissions are reduced by 7.14% for 10% SAME, 8.57% for 20% SAME, and 10.71% for 30% SAME when the maximum load is applied. The same trend is noticed in the results of Refs.^42–44^.

**Figure 9:**
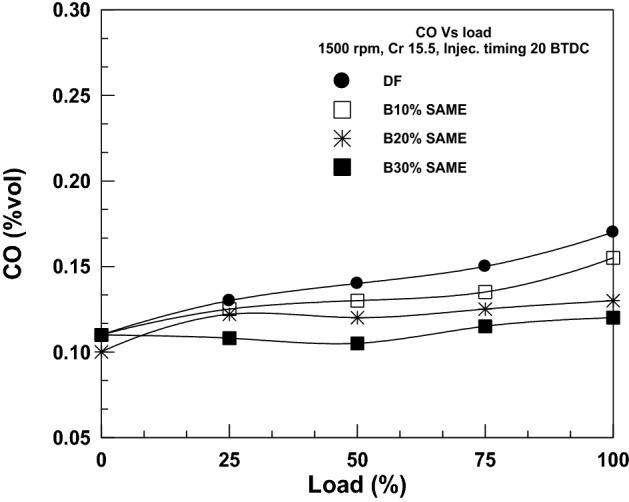
Change of CO with brake power.

The correlation between CO emissions and load for various SAME/DF mixtures is shown in Fig. 9. For all tested fuels, carbon monoxide emissions are found to be lowest at light load (0 KW) and greatest at full load (3.7 KW). Adding biodiesel reduced CO_2_ emissions because more of the fuel was burned with the additional oxygen supply in the engine’s combustion chamber. The experimental findings indicated a decrease in CO emissions, which is a positive finding. CO emissions for SAME blends decreased by 6.67%, 11.34%, and% when using 10, 20, and % SAME, respectively, compared to DF at full load.

**Figure 10:**
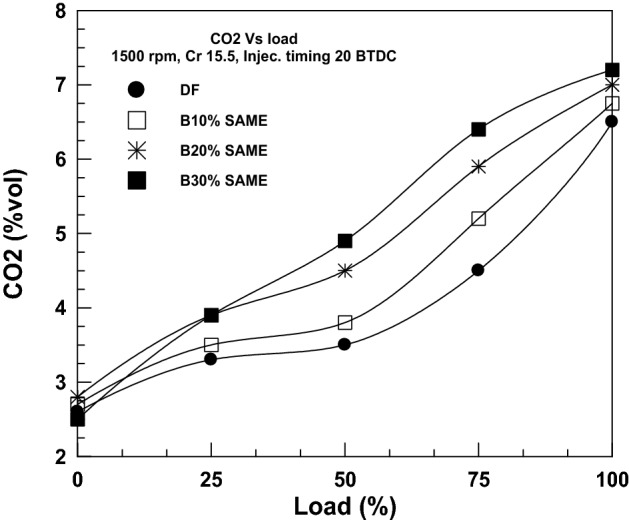
Change of CO_2_ with brake power.

CO_2_ emissions, mostly from internal combustion engines, are also a key contributor to climate change. Figure 10 shows CO_2_ emissions for various engine loads and fuel mixtures. Increasing engine load with any given fuel mixture results in an increase in CO_2_ emissions. Adding more biodiesel to the fuel mixture increases carbon dioxide emissions. CO_2_ emissions rose with an increasing SAME ratio at maximum engine load. The increased oxygen content in biodiesel combines with CO to generate CO_2_, which accounts for the increased CO_2_ emissions of mixed fuels. Plants can use the carbon dioxide put into the air to make food and fuel, like seeds and oil, through a process called photosynthesis. In accordance with the biodiesel life cycle, the carbon dioxide released during burning has the same environmental impact as the photosynthesis of carbon dioxide that is reprocessed by plants, trees, and even subsequent algal crops^41^.

**Figure 11:**
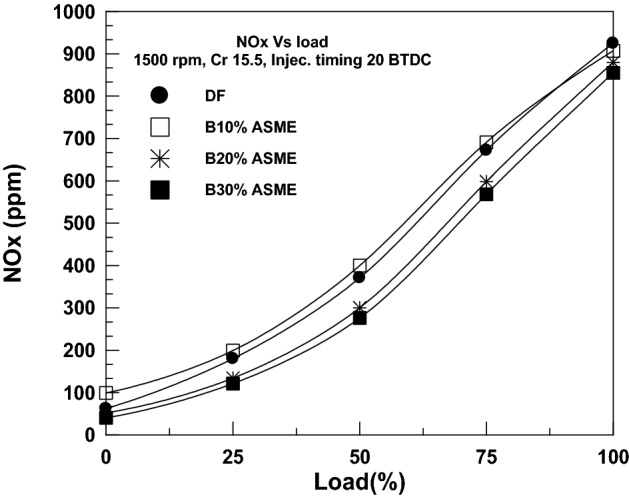
Change of CO_2_ with brake power.

Figure 11 demonstrates how NOx emissions rise because combustion temperatures rise as engine load increases. The NOx emission for 30% SAME at no load (0 KW) was 62 ppm, while it was 925 ppm at 3.7 KW. As SAME biodiesel’s blending ratio rises, NOx emissions also rise because the amount of oxygen in biodiesel increases. Results showed a 2.92%, 6.08%, and 8.18% increase in NOx emissions for 10% SAME, 20% SAME, and 30% SAME, respectively, as compared to DF. These results are similar to the results reported by many researchers, such as Refs.^45,46^.

NOx emissions are one of the most critical aspects of biodiesel and its mixture’s emissions. It is the main focus of the majority of biofuel researchers. Figure 11 depicts the rise in NOx emissions caused by higher combustion temperatures as engine load increases. SAME had NOx emissions of 62 ppm at no load (0 kW) and 925 ppm at 3.7 kW. Due to the rise in the oxygen content of biodiesel, NOx emissions increase as the blending fraction of SAME biodiesel rises. The total load results indicated a rise in NOx emissions of 2.92%, 6.08%, and 8.18% for 10% SAME, 20% SAME, and 30% SAME, respectively, compared to DF.

### The impact of compression ratio on the performance and emissions parameters

To further examine the impact of changing compression ratio on the same characteristics of combustion and emissions addressed in “Examination of the performance and emissions parameters” section, we chose two more compression ratios to test (14.5:1 and 16.5:1) in addition to the engine’s usual compression ratio (15.5:1). The standard full load condition is picked because of the lower A/F ratio stated in the event of full load; thus it is the best point to distinguish among the varied scope.

**Figure 12:**
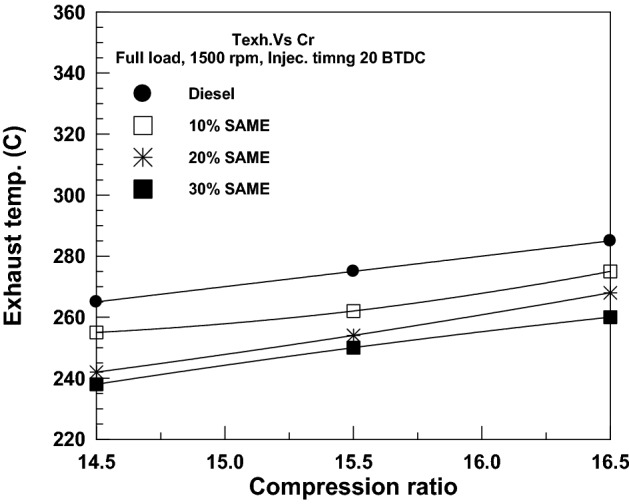
Change of EGT with compression ratio.

The influence of VCR on EGT for diesel and SAME mixes is seen in Fig. 12. Increasing the compression ratio raises cylinder pressure and temperature, putting more thermal stress on the engine and thereby increasing the exhaust gas temperature (EGT).

**Figure 13:**
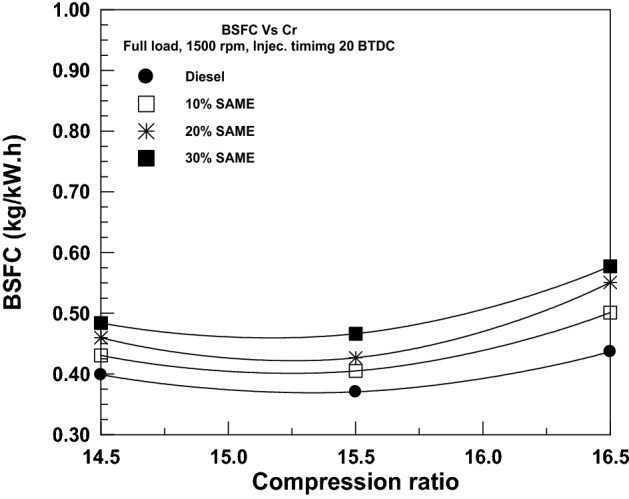
Change of BSFC with a compression ratio.

The influence of VCR on the BSFC for diesel and SAME mixes is seen graphically in Fig. 13. Increases in compression ratio improve BSFC because less air is let into the engine, leading to greater fuel consumption. The BSFC score is the highest for a mix with 30% SAME and the lowest for a mix with DF.

**Figure 14:**
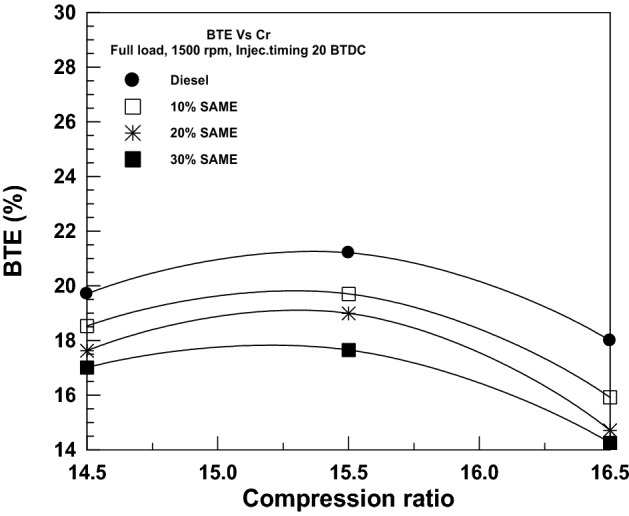
Change of BTE with a compression ratio.

The correlation between BTE and compression ratio is seen in Fig. 14. While increasing the compression ratio from 14.5/1 to 15.5/1, the BTE increases for all fuels studied. However, when increasing the compression ratio from 15.5/1 to 16.5, the BTE begins to decline as a result of the higher heat load and friction experienced by the engine. BTE measurements indicated a decrease for All SAME mixes compared to pure DF.

**Figure 15:**
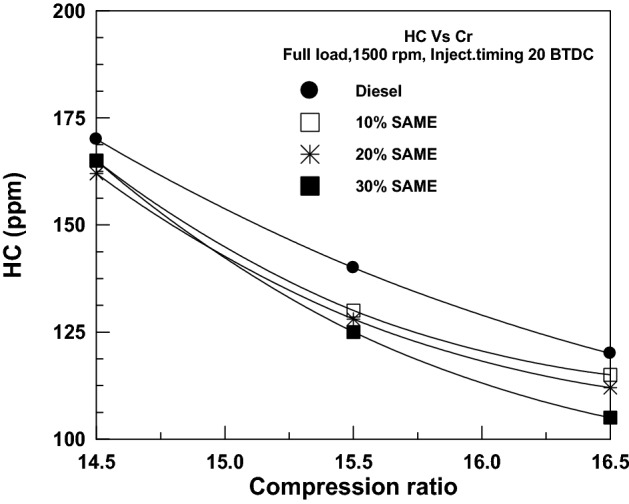
Change of HC with compression ratio.

Hydrocarbon (HC) emissions are seen clearly in Fig. 15, where a rise in compression ratio results in a steep drop in emissions due to a shorter combustion period. HC emissions decreased as the SAME amount rose due to an important factor: algal fuel naturally includes oxygen in its fuel structure, which accelerates oxidation and causes full burning^47,48^. SAME levels of 10%, 20%, and 30% were shown to reduce HC significantly compared to DF levels at the start.

**Figure 16:**
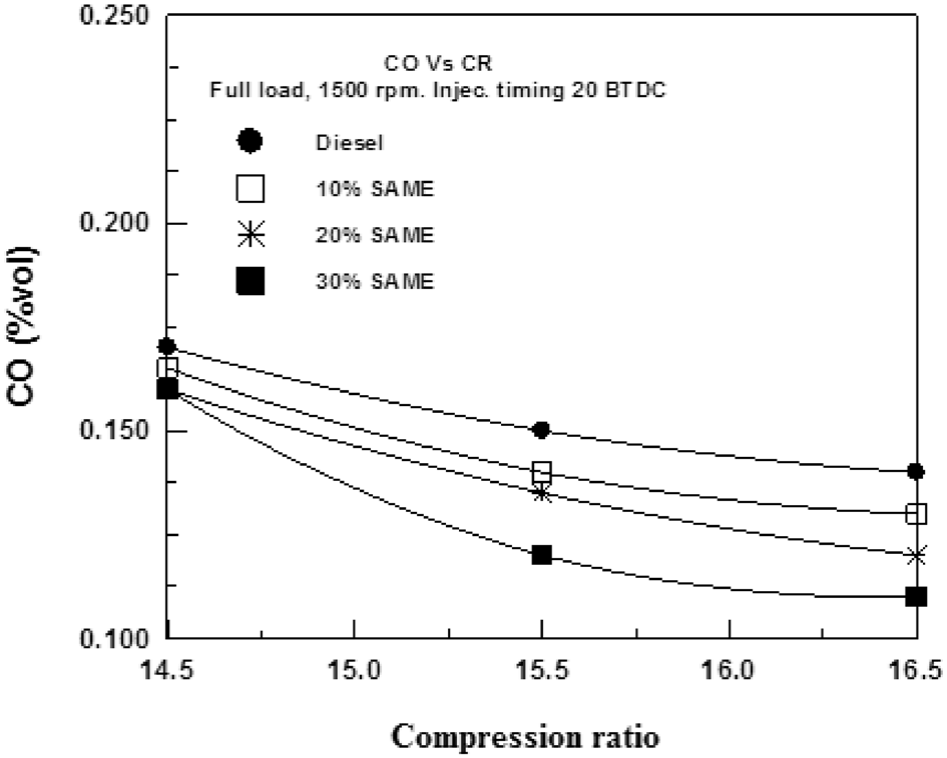
Change of CO with compression ratio.

Figure 16 depicts the effect of the VCR on CO emissions. CO levels dropped from 14.5/1 to 16.5/1 as the compression ratio increased; this was because the shorter burn period resulted in less CO_2_ being produced. However, when SAME mixes (at 10%, 20%, and 30%) were employed instead of DF, CO emissions dropped significantly.

**Figure 17:**
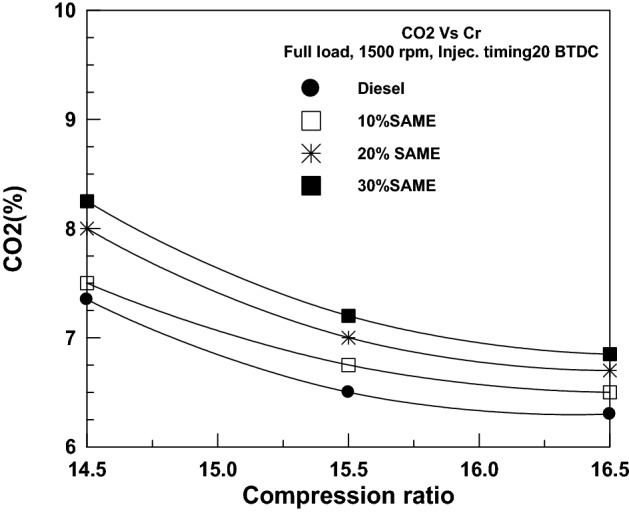
Change of CO_2_ with a compression ratio.

A visualization of VCR's effect on CO_2_ emissions is shown in Fig. 17. As was previously indicated, a higher compression ratio results in lower CO_2_ emissions. The abundant oxygen in the SAME content aids in oxidizing CO to CO_2_. The net result is a decrease in CO emissions and an increase in CO_2_ emissions. Carbon dioxide emissions are lowest for DF, then at 10% SME, 20% SAME, and finally at 30% SAME.

**Figure 18:**
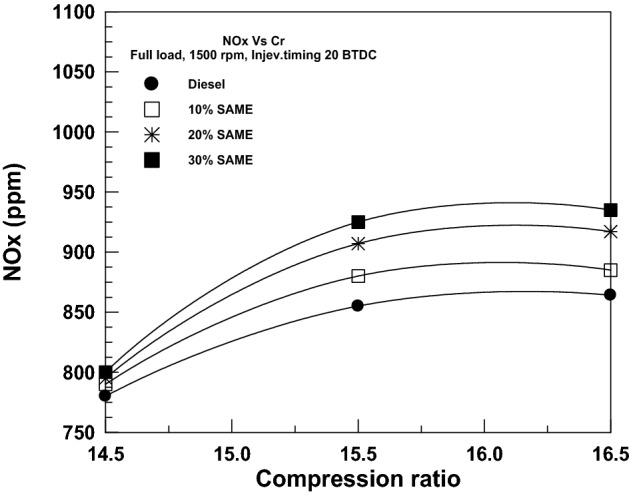
Change of NO_x_ with compression ratio.

Figure 18 shows the impact of VCR on NOx emissions from DF and SAME mixes. The oxygen content in the structure of biodiesel has two effects: first, it lowers carbon emissions; second, it provides an additional source of oxygen from which NOx emissions are produced. Adding biodiesel to DF decreases carbon emissions but increases NOx levels, creating a so-called trade-off connection. As shown in Fig. 18, the NOx level is highest for the 30% SAME and lowest for the DF. The findings of Ref.^49^ come to fit the current results.

## Conclusions

- A higher BSFC and lower BTE were seen when SAME mixes were added to DF.
- CO and HC emissions went down a lot, while CO_2_ and NOx emissions increased slightly.
- As the compression ratio rises, the BTE rises, whereas the BSFC decreases.
- When the compression ratio goes up, all carbon emissions noticeably decrease, as well as the cylinder pressure and temperature go up, which increases NOx.
- The data shows that the SAME blending ratio of 30% is the optimal compromise.
- When SAME biodiesel is used, NOx emissions go up. Adding nanoparticles is recommended, which may help reduce this effect and improve performance simultaneously.

The current technique could be applied to a variety of physical and technical challenges in the future^50–57^.

## Data Availability

All data generated or analyzed during this study are included in this published article.
